# Editorial: The “GEnomics of MusculoSkeletal traits TranslatiOnal NEtwork” (GEMSTONE)

**DOI:** 10.3389/fendo.2022.1110170

**Published:** 2022-12-23

**Authors:** J.H. Tobias

**Affiliations:** ^1^ Musculoskeletal Research Unit, Department of Translational Health Sciences, Bristol Medical School, University of Bristol, Bristol, United Kingdom; ^2^ Medical Research Council (MRC) Integrative Epidemiology Unit, Department of Population Health Sciences, Bristol Medical School, Faculty of Health Sciences, University of Bristol, Bristol, United Kingdom

**Keywords:** genetics, osteoporosis, bone mineral density, mouse model, zebrafish, DXA (dual x-ray absorptiometry)

The Genomics of Musculoskeletal traits Translational Network (GEMSTONE), funded by a European Cooperation in Science and Technology (COST) Action “aims to bring together multiple disciplines currently active in the field of musculoskeletal research under a coordinated effort, which allows translating the emerging wealth of genetic discoveries into palpable clinical applications that can help setting the ground for personalized medicine” https://cost-gemstone.eu/about. This network primarily focuses on the common age-related musculoskeletal disorder, osteoporosis. One of the main outputs of this network has been to produce six review articles describing the state of the art in osteoporosis genetics research, as summarised below (five of these papers appeared in the GEMSTONE research topic, and a further paper in Rising stars in Bone Research 2021).

The paper by Koromani et al. describes the origins, rationale, organisation and prospects for GEMSTONE. Over the last decade, genome wide association studies (GWAS) studies have led to the identification of many new genetic influences on intermediary traits for osteoporosis such as bone mineral density (BMD). However, the function and mechanisms by which such genetic factors affect these traits is unknown. GEMSTONE brings together multidisciplinary expertise to identify these mechanisms, and ultimately translate this understanding for the benefit of patient care. In order to achieve these goals, GEMSTONE has brought together a range of experts, who are organized across six working groups (WGs) namely (Koromani et al.) “Study populations and expertise groups” (Trajanoska and Rivadeneira), “Phenotyping” (Foessl et al.), “Monogenic conditions- human knockout (KO) models” (Kague et al.), “Functional investigations” (Formosa et al.), “Bioinformatics” and (Rauner et al.) “Translational outreach”. These working groups have aimed to describe the state-of-the-art in these different areas; where complete this has led to papers presented herein.

The paper by Trajanoska and Rivadeneira further sets the scene for GEMSTONE by summarizing the achievements of genetic studies in the bone field to date, and discussing how these could be translated for the benefit of patients. Many mutations are now recognised to cause a range of monogenic bone disorders including Osteogenesis Imperfecta and Osteopetrosis, as well as several sclerosing bone disorders. The latter include sclerosteosis, where identification of the underlying genetic mutation led to development of romosozumab, a new monoclonal antibody treatment for osteoporosis *via* sclerostin inhibition. On the other hand, GWAS studies of complex traits such as BMD have identified many hundreds of genetic influences, of which some are related to known bone regulatory pathways, whereas others are entirely novel. These mechanisms might provide drug targets for further treatments for osteoporosis, either in the form of novel therapies, or by providing a rationale for re-purposing drugs already used for other conditions. Understanding the genetic basis of osteoporosis could also lay the foundation for pharmacogenomic studies to identify patients who respond particularly well or poorly to a specific treatment, or are more susceptible to adverse effects. Such data can also provide the basis for polygenic risk scores for osteoporosis, intended to complement other methods for individualised fracture risk assessment.

(1) The paper by Foessl et al., on behalf of WG2, reviews the different methods used for phenotyping the human skeleton, as well as how these have been applied to mammalian animal models and zebrafish. The comprehensive description of clinical phenotyping methods comprises (i) features that should be sought from clinical history taking and examination, (ii) widely available imaging and laboratory tests such as X-rays, DXA and bone turnover markers, (iii) patient reported outcome measures from questionnaires, and (iv) more specialized methods more commonly used in the context of research studies such as HRpQCT and trans-iliac bone biopsy. The detailed review of imaging methods divides these into 2D and 3D modalities, comparing applications between humans, rodents and zebrafish. A number of methods for *ex-vivo* analysis of bone tissue also exist including histomorphometry, compositional bone matrix analysis using backscattered electron microscopy imaging, and immunohistochemistry.

The paper by E Kague et al. (published in Rising Stars in Bone Research 2021), represents a further output of WG2, by illustrating how analysis of cranial sutures in zebrafish, which unlike in humans remain open during lifetime, can be used in functional validation of osteoporosis-related genes. The rationale for this approach stems from the finding that, as well as several known BMD loci, a recent GWAS of skull BMD identified a number of novel genetic factors. Moreover, a significant overlap was identified between genetic influences on skull BMD, and craniosynostosis, a rare congenital disorder involving premature fusion of the skull sutures. The authors subsequently evaluated the impact of targeting novel genes identified from their skull BMD GWAS in zebrafish by CRISPR/Cas9 to produce loss of function. In all cases, targeting these genes led to marked abnormalities in suture growth, confirming the utility of certain skeletal phenotypes in zebrafish in functional validation of osteoporosis genes identified from GWAS studies.

The paper by Formosa et al., on behalf of the WG3, examines the utility of monogenic low and high bone mass disorders for identifying novel therapeutic targets for osteoporosis. This paper provides a comprehensive description of monogenic disorders associated with alterations in bone mass, dividing these according to whether BMD is either increased or decreased. A number of steps are involved in identifying new disorders of this type, including careful clinical phenotyping, and emerging bioinformatic approaches to evaluating whole exome/whole genome sequencing data. Cell based, rodent and zebrafish models are widely used for functional validation of putative genetic mutations. Ultimately, findings from such studies can be used to identify therapeutic targets for both osteoporosis and rare bone diseases, as exemplified by romosozumab for the treatment of osteoporosis discussed above.

Finally, the paper by Rauner et al., on behalf of WG4, describes the different methods used for identifying causal genes underlying association signals identified in human genetic studies. This is pertinent for analysing both GWAS studies of traits such as BMD, and putative genetic mutations associated with rare skeletal disease. In GWAS studies, a Mendelian Randomisation framework can be used to link a genetic association signal to an mRNA expression quantitative trait locus eQTL (eQTL) or protein expression quantitative trait locus (pQTL) related to a specific gene. Another important tool is the annotation of genetic sequences in coding and non-coding regions. Whereas monogenic disorders most commonly arise from altered protein coding, the great majority of signals identified in GWAS studies are in non-coding regions. Genetic variation in non-coding regions can affect gene transcription *via* a wide range of mechanisms, including altered DNA methylation, binding of transcriptional enhancers and repressors, and non-coding RNAs (ncRNAs) including microRNAs (miRNAs). This paper also describes the various laboratory methods used for functional follow up of genetic associations. 17 research teams from the GEMSTONE consortium provide a range of cellular resources derived from different tissues represented in bone, from distinct species. In terms of animal models, though the mouse has been the most widely studied historically, zebrafish are being increasingly used as they offer certain advantages such as more rapid throughput. Animal models enable evaluation of the impact of over-expression or deletion of a given gene, either at the level of the whole organism or limited to bone using the Cre/loxP recombination system. As well as perturbation of the entire gene, gene-editing technologies enable the insertion of specific genetic variants in order to evaluate the impact of specific coding changes linked to rare skeletal disorders. To bring together these various resources in order to interrogate any given genetic signal of interest, the musculoskeletal knowledge portal (MSK-KP) has recently been created by the International Federation of MSK Research Societies (IFMRS) [https://msk.hugeamp.org/].

Together, these six papers provide a comprehensive description of the state-of-the art in genetic research in osteoporosis and related disorders, highlight the important directions and challenges for future research, and will be of interest to researchers and clinicians alike.

## Author contributions

The author confirms being the sole contributor of this work and has approved it for publication.
